# The Impacts of Ramadan Intermittent Fasting on Saliva Flow-Rate and Metabolic Data: A Systematic Review

**DOI:** 10.3389/fnut.2022.873502

**Published:** 2022-04-06

**Authors:** Amira Besbes, Mehdi Khemiss, Nicola Bragazzi, Helmi Ben Saad

**Affiliations:** ^1^Laboratory Research of Medical and Molecular Parasitology and Mycology, LR12ES08, Faculty of Pharmacy, University of Monastir, Monastir, Tunisia; ^2^Unit of Microbiology, Faculty of Dental Medicine, University of Monastir, Monastir, Tunisia; ^3^Department of Dental Medicine, Fattouma Bourguiba University Hospital, University of Monastir, Monastir, Tunisia; ^4^Laboratory for Industrial and Applied Mathematics (LIAM), Department of Mathematics and Statistics, York University, Toronto, ON, Canada; ^5^Department of Health Sciences, Postgraduate School of Public Health, University of Genoa, Genoa, Italy; ^6^NIHR Leeds Musculoskeletal Biomedical Research Unit, Section of Musculoskeletal Disease, Chapel Allerton Hospital, Leeds Institute of Molecular Medicine, University of Leeds, Leeds, United Kingdom; ^7^Research Laboratory “Heart Failure, LR12SP09”, Faculty of Medicine, Hospital Farhat Hached, University of Sousse, Sousse, Tunisia

**Keywords:** cortisol, oral health, melatonin, Ramadan fasting, salivary biomarkers, salivary flow rate

## Abstract

The aim of this systematic review was to report the impacts of Ramadan intermittent fasting (RIF) on salivary flow-rate (SFR) and metabolic parameters. A thorough literature search was carried out using the databases *PubMed* and *Scopus* from their inception up to 15 July 2021. The Boolean connectors used in *PubMed* were (Saliva [Mesh] AND Fasting [Mesh]). The same keywords were used in *Scopus*. Inclusion criteria were defined using PICOS. The research included all original studies involving “healthy” adults and published in English. Methodological quality assessment was performed utilizing the Joanna Briggs Institute Critical Appraisal Tool, which allows attributing scores from 1 to 11 to the selected studies. Two authors carried out the literature search, study selection, and data extraction. Differences on issues were resolved by a third author if necessary. The systematic review protocol was registered within the “Open Science Framework” (Doi: 10.17605/OSF.IO/DE7BH). Six articles met the inclusion criteria. All studies were heterogeneous and had a high score of bias and several methodological differences. The following parameters were collected: SFR, melatonin, cortisol, glucose, immunoglobulin A (IgA), uric-acid, alkaline phosphatase (ALP), and aspartate aminotransferase (AST). The SFR decreased by 10% during Ramadan in fasting subjects. The circadian pattern of melatonin remained unchanged during Ramadan, but melatonin levels dropped significantly from baseline. The salivary cortisol levels were unchanged or increased during Ramadan. The salivary glucose levels were decreased. ALP increased significantly, whilst uric-acid and AST decreased significantly. Salivary IgA decreased during the last week of Ramadan. To conclude, there is a trend toward a decrease in SFR and the content of the majority of the biomarkers investigated, with the exception of ALP and uric-acid. These changes cannot be easily attributed to any single factor (hydration status, dietary habits, physical activity, or hygiene habits).

**Systematic Review Registration:** [https://osf.io/de7bh/], identifier [Doi: 10.17605/OSF.IO/DE7B].

## Introduction

Human saliva is a biofluid produced and secreted by the major and minor salivary glands (1). The major salivary glands are the parotid, submandibular, and sublingual glands, responsible for more than 90% of salivary secretions, and the minor glands are distributed throughout the oral mucosa surfaces (1). Saliva plays an essential role in oral cavity maintenance and functionality (1), and it represents a mirror reflecting both oral and systemic health (2). Salivary secretions are composed of water, electrolytes, and several biomolecules, including proteins, enzymes, exosomes, nuclear acids, hormones, and cellular components (2). Many studies have demonstrated that the composition of saliva varies depending on the type of stimulation (2), the short-term acute mental stress (3), the taste and smell (4), and the daily and seasonal circadian rhythms (5). Hence, recurrent circadian fasting during Ramadan [i.e., Ramadan intermittent fasting (RIF)] may modify the salivary parameters.

Ramadan is the ninth month of the Muslim lunar calendar and it lasts 29 or 30 days depending on the actual observation of the moon’s crescent (6). The synodic nature of the Muslim calendar means that Ramadan occurs 10–11 days earlier each Gregorian year, migrating across all four seasons over approximately a 33-year cycle (6). Therefore, the fasting daytime duration can vary accordingly with longer fasting durations during summer. At any time point, the geographical situation will have an impact on the daylight. The higher the latitude is, the longer the fasting duration will be (7). Recurrent circadian fasting during Ramadan is practiced by around two billion Muslims every year (8), and healthy adult Muslims are asked to refrain from eating and drinking during this month between *Sahur* (dawn meal just before the start of fast) and *Iftar* (sunset meal marking the end of the fast) as a religious duty (6). Since food and water intake takes place from sunset to dawn, this modification in Muslims’ lifestyle for 1 lunar month may have an impact on oral health. A Muslim may be exempt from fasting during Ramadan (DR) for several reasons, including pregnancy, breastfeeding, diabetes mellitus, and mental disability, however; despite these exemptions, many Muslim patients with chronic medical conditions still choose to fast (9).

Several systematic reviews have studied the effects of RIF on general health (10–12), notably on the immune system (13), cardiovascular function (14), dietary intake and body composition or weight (15, 16), glycemic control (17), kidney function (18), and sleep (19). However, to the best of the authors’ knowledge, no previous systematic review has investigated the impacts of RIF on salivary secretion [e.g., salivary flow-rate (SFR)] and metabolic parameters such as cortisol, glucose, melatonin, and uric-acid. The aim of this paper was therefore to systematically review the impacts of RIF on SFR and saliva metabolic parameters.

## Methods

### Protocol and Eligibility Criteria

The systematic review protocol was registered within the “Open Science Framework” (OSF, DOI 10.17605/OSF.IO/DE7BH). This systematic review followed the “Preferred Reporting Items for Systematic Reviews and Meta-Analyses” (PRISMA) guidelines (20). The inclusion criteria were formulated based on the following PICOS tool questions (21): ***P*** (population) = healthy Muslim adults willing to fast DR; ***I*** (intervention/exposure) = exposure to RIF; ***C*** (Comparison): DR and outside Ramadan [i.e., before-Ramadan (BR) and after-Ramadan (AR)]; ***O*** (Outcome): SFR and saliva metabolic parameters; and ***S*** (Study design): all original articles written in English. No restrictions were applied in terms of study design, setting, country, or period. Publications not in compliance with the purpose of this systematic review as well as those not representing original research (i.e., reviews, editorials, qualitative papers, case reports, case series, and letters to editors) were not included.

### Literature Search

An online literature search was performed using two databases: *PubMed* and *Scopus* from their inception up to 15 July 2021. For *PubMed*, the search was carried out using a strategy employing the combination of the following two “Medical Subject Headings” (MeSH) terms: *Saliva* AND *Fasting*. As for *Scopus*, the previous two terms were searched for in the article titles, abstracts, and/or keywords. In addition, the reference lists of the included articles were checked. All the authors involved in this review agreed on the articles to be included in this systematic review.

### Study Selection

**FIGURE 1:**
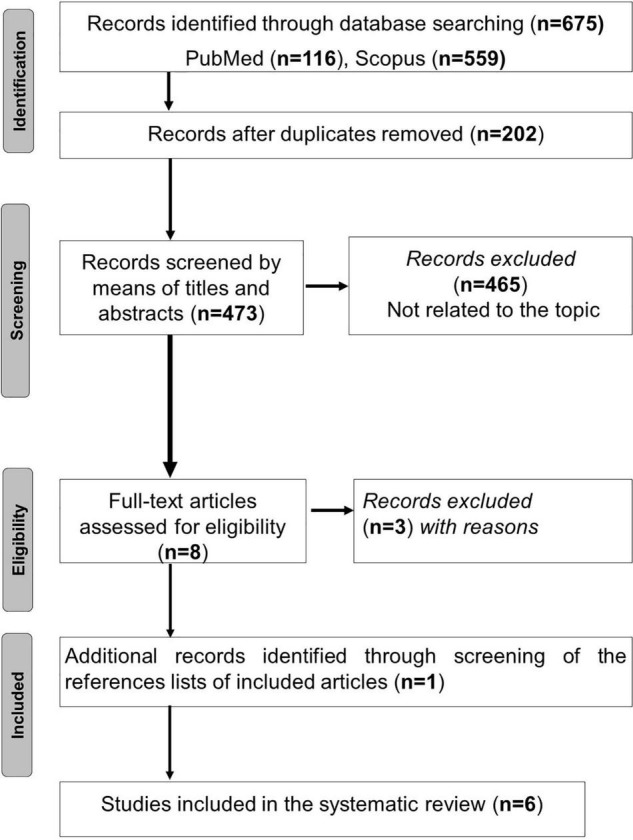
Study flowchart.

The process of articles selection is outlined in Figure 1. Duplicate articles were eliminated using End-Note X9 library. Titles of the remaining articles were independently appraised during the initial online literature search for studies by two of the authors (*AB* and *MK* in the authors’ list) to check for their relevance to the searched topics. Abstracts of these titles were then read to determine if the studies met the inclusion criteria. The studies whose abstracts met the inclusion criteria were then read in full-text format to determine their eligibility and therefore retention. Two authors (*AB* and *MK* in the authors’ list) conducted the study selection process for this review, with discrepancies being checked by a third author (*HBS* in the authors’ list), if necessary.

### Data Extraction

Data from the retained studies were extracted using a format including the population, the parameters being investigated, the periods during which the parameters were collected, and the significant findings. Data were extracted, reviewed, and analyzed by two authors (*AB* and *MK* in the authors’ list). Extracted data were then verified by a third author (*HBS* in the authors’ list). Discrepancies in data collection were resolved through discussion.

### Methodological Quality Assessment

Methodological quality assessment was performed using the Joanna Briggs Institute (JBI) critical appraisal tool, precisely the checklist for cohort studies (https://joannabriggs.org/last visit: 4 March 2022). The checklist appraises the following areas: recruitment, exposure measurement, reliability of exposure measurement, confounding factors identified, strategies to deal with confounding factors, participants free of outcome at the onset of the study, validity and reliability of outcome measurement, follow-up timeframe reported, follow-up completion, strategies utilized to deal with incomplete follow-up, and appropriate statistical analysis. The checklist included the following 11 items: ***1.*** Were the two groups similar and recruited from the same population? ***2.*** Were the exposures measured similarly to assign people to both exposed and unexposed groups? ***3.*** Was the exposure measured in a valid and reliable way? ***4.*** Were the confounding factors identified? ***5.*** Were the strategies to deal with confounding factors stated? ***6.*** Were the groups/participants free of the outcome at the start of the study (or at the moment of exposure)? ***7.*** Were the outcomes measured in a valid and reliable way? ***8.*** Was the follow-up time reported and sufficient to be long enough for outcomes to occur? ***9.*** Was follow-up complete, and if not, were the reasons for loss to follow-up described and explored? ***10.*** Were the strategies to address incomplete follow-up utilized? and ***11.*** Was the appropriate statistical analysis used? These items are scored as either yes, no, unclear, or not applicable. Two reviewers (*AB* and *MK* in the authors’ list) independently scored the retained studies, with discrepancies being resolved through discussion. If discrepancies could not be resolved through discussion, a third author (*HBS* in the authors’ list) intervened to reach consensus. The risk of bias in the studies was judged to be low (“yes” scores > 70%), moderate (50 ≤ “yes” scores between ≤ 69%), and high (“yes” scores < 49%) (22).

## Results

### Search Results

The search process yielded 675 articles, of which 202 were duplicated. Among the 473 remaining papers, 465 were excluded based on the title and the abstract. When screening the references lists of the remaining eight articles (23–30), one additional paper was added (31). After assessing full-text articles for eligibility, three articles were excluded (23–25). Consequently, six articles were retained (26–31). The search results are presented in Figure 1.

### Methodological Quality Assessment Results

The retained six studies were assessed for methodological quality (Table 1). All the studies have a high score of bias (i.e., final score ranging from 9.1 to 36.4%). *Items 2*, *5*, and *10* were rated as not applicable for all the studies. No study reported data regarding *items 4, 7*, and *11*. Six (26–31), five (26–29, 31), four (27–29, 31), one (29), and one (31) studies included information regarding *items 6*, *8*, *3, 9*, and *1*, respectively.

**TABLE 1:** Quality scoring of the retained articles according to Joanna Briggs Institute critical appraisal checklist.

First author	Reference	1	2	3	4	5	6	7	8	9	10	11	Score (%)	Study risk of bias
Bahammam	(26)	N	N/A	N	N	N/A	Y	N	Y	N/A	N/A	N	18.2	High
Sariri	(31)	N	N/A	Y	N	N/A	Y	N	Y	N/A	N/A	N	27.3	High
Develioglu	(27)	N	N/A	Y	N	N/A	Y	N	Y	N/A	N/A	N	27.3	High
Khaleghifar	(28)	N	N/A	Y	N	N/A	Y	N	Y	N/A	N/A	N	27.3	High
Dehaghi	(30)	N	N/A	N	N	N/A	Y	N	N	N/A	N/A	N	9.1	High
Al-Rawi	(29)	N	N/A	Y	N	N/A	Y	N	Y	Y	N/A	N	36.4	High

*N, no; N/A, not applied; U, unclear; Y: yes. Item 1. Were the 2 groups similar and recruited from the same population? Item 2. Were the exposures measured similarly to assign people to both exposed and unexposed groups? Item 3. Was the exposure measured in a valid and reliable way? Item 4. Were the confounding factors identified? Item 5. Were the strategies to deal with confounding factors stated? Item 6. Were the groups/participants free of the outcome at the start of the study (or at the moment of exposure)? Item 7. Were the outcomes measured in a valid and reliable way? Item 8. Was the follow-up time reported and sufficient to be long enough for outcomes to occur? Item 9. Was follow-up complete, and if not, were the reasons for loss to follow-up described and explored? Item 10. Were the strategies to address incomplete follow-up utilized? Item 11. Was the appropriate statistical analysis used?*

### Study Selection and Characteristics

Table 2 exposes the main characteristics and methodological points of the retained studies. The latter were published between 2004 (26) and 2020 (29, 30). The studies were conducted in Saudi Arabia (26), Turkey (27), United Arab Emirates (29), and Iran (28, 30, 31). The study design was not reported in three studies (27–29). In the remaining studies, three designs were applied: observational design with repeated measures (26), case-control (31), and descriptive analytical research design (30). All the studies (26–31) opted for convenience samples. The Ramadan year was omitted in three studies (28, 30, 31). Only one study (29) mentioned the number of fasting days DR. Only one study (28) reported the average ambient temperature, which was around 15°C. Only three studies reported the mean fasting duration [i.e., 12 h (26), 15 h (29), 17 h (27)]. No study reported data with regard to the Ramadan season, the average ambient pressure, or the average ambient humidity.

**TABLE 2:** Main characteristics and methodology points of the published studies aiming to evaluate the impacts of Ramadan intermittent fasting (RIF) on saliva parameters.

First author (ref)	Bahammam (26)	Sariri (31)	Develioglu (27)	Khaleghifar (28)	Al-Rawi (29)	Dehaghi (30)
Town (country)	Riyadh (Saudi Arabia)	Tehran (Iran)	Istanbul (Turkey)	Rasht (Iran)	Sharjah (United Arab Emirates)	Ahvaz (Iran)
Yr of publication	2004	2010	2012	2017	2020	2020
Ramadan Yr	2002	2007	2012	NR	2017	2018
Study design	Observational study with repeated measures	Case-control study	NR	NR	NR	Descriptive and analytical study
Evaluation sessions’ number	2 weeks BR 1st week of R 3rd week of R	1–9 first days of R 10–20 second days of R 21–29 last days of R 7th day AR	BR (1 week BR) 25th day of R (last week of R)	1 day BR (used as control) 25 day of R Last day of R	1 week BR 28 day of R	1 week BR 3rd week of R
Inclusion criteria	No regular medications No alcohol	Healthy Males Students	Healthy Males	Healthy non-smokers Male Healthy teeth Healthy mouth No oral disease No internal disease	Overweight/obese Muslims	Nurses Fasting during the study period
Non-inclusion and exclusion criteria	Sleep complaints Smoking Addiction to caffeinated beverages	Severe infection Oral and/or dental diseases	Acute diseases Chronic disease Medication-use	NR	Diabetes-mellitus Endocrine disease Cardiovascular diseases Medication-use Pregnancy Bariatric surgery Weight management program	Hearing impairment Headache Head surgery Psychiatric shock- last 6 months Cardiovascular disease Metabolic disorders Diabetes-mellitus Job experience <1 year
Participants’ number (M/F)	8 (NR/NR)	Fasting group: 30 (30/0) Control group: 30 (30/0)	24 (19/5)	35 (35/0)	57 (40/17)	75 (39/36)
Age (years)	31.8 ± 2.0*^a^*	24.2 ± 2.3*^a^*	35.9 ± 11.1*^a^* 20–59*^b^*	30–50*^b^*	38 ± 11*^a^*	Hospital 1: 36.3 ± 8.8*^a^* (M), 35.5 ± 7.6*^a^* (F) Hospital 2: 37.2 ± 9.1*^a^* (M), 37.7 ± 6.2*^a^* (F)
Weight (kg)	NR	NR	77.2 ± 1.4*^a^* (BR) 76.0 ± 11.6*^a^* (DR)	NR	88.3 ± 16.2*^a^* (BR) 86.7 ± 15.7*^a^* (DR)	NR
BMI (kg/m^2^)	25 ± 2.2*^a^*	NR	25.5 ± 3.5*^a^* (BR) 25.1 ± 3.5*^a^* (DR)	NR	29.9 ± 5.02*^a^* (BR) 29.4 ± 4.9*^a^* (DR)	NR
Collected saliva parameters and time	Melatonin 3 times (08:00; 16:00; 00:00)	SFR (= time required to collect 1 ml of saliva in 1 minute) Glucose 3 samples at mid-day (after 6 h of fasting)	Immunoglobulin A Saliva samples were taken after a 12-h overnight fast BR and 12-h after the last meal during-R	Uric-acid ALP AST Saliva sample at noon (after 8 h of fasting)	Cortisol Fixed times of the day (11:00–13:00).	Cortisol 2 times (06:00, 16:00)
Type of saliva (volume)	NR (5 mL)	Timed (2–5 min) unstimulated saliva (3 mL)	Timed (5-min) unstimulated saliva (NR)	Unstimulated saliva (3 mL)	Unstimulated saliva (NR)	NR (2 mL)
Used equipment	Highly sensitive radioimmunoassay kit	Enzymatic assay glucose kit	Behring Nephelometer	Enzymatic uric-acid assay kit Kits for assay of AST and ALT	NR	Cobase radioimmunoassay kit with electro-chemiluminescence
Participant instructions	Rinse the mouths with water before collection Avoid coughing or throat clearing into the collection tube Avoid consuming caffeine and substances containing melatonin or melatonin precursors	Gargling the mouth with about 5 ml of distilled water for 2 min	Mouth rinsed by distilled water	Gaggling the mouth with about 5.0 ml of distilled water for about 1 minute.	Avoid eating, drinking, and smoking Not to practice oral hygiene at least 1 h before No special dietary recommendations Continue a regular diet during non-fasting hours. Do not alter the habitual physical exercise levels BR or DR	In the morning brushing, eating, drinking and/or smoking was forbidden before taking the saliva sample.
Other details	Fixed daytime working hours Regular sleep-wake schedule during week-ends Same type of work, tasks, and working hours during the study period	Mouth and teeth were examined before saliva collection	The content of the participants’ diets was similar BR and DR No URTIs during the study period	NR	No sleep problems Regular sleep/wake schedule No participant practiced fasting as routine, and voluntary rituals BR	NR

*ALP, alkaline phosphatase; AR, after-Ramadan; AST, aspartate amino-transferase; BMI, body-mass-index; BR, before-Ramadan; DR, during-Ramadan; F, females; h, hour; Ig, immunoglobulin; M, males; NR, not-reported; R, Ramadan; SFR, salivary flow-rate; URTIs, upper respiratory tract infections; Yr, year. Data were: ^a^Mean ± SD; ^b^Minimum-maximum.*

The number of evaluation sessions was two (27, 29, 30), three (26, 28), and four (31). Five studies (26–30) opted for a session BR with different periods applied [i.e., 1 day BR (28), 1 week BR (27, 29, 30), 2 weeks BR (26)]. Only one study opted for a session AR (i.e., 7 days AR) (31). The number of sessions DR was one (27, 29, 30), two (26, 28), and four (31), and different periods were retained [i.e., 1 week (26), first 10 days (31), 10–20 second days (31), third week (26, 30), 21–29 last days (31), 25th day of Ramadan (27, 28), last day of Ramadan (28, 29)].

Two-hundred twenty-nine participants fasting DR were included. The sample sizes varied from 8 (26) to 75 (30). Three studies included mixed population of males and females (27, 28, 30), two studies included only males (28, 31), and the participants’ sex was not reported in one study (26). Four studies included healthy participants (26–28, 31), one study involved both overweight and obese participants (29), and one study omitted to report the health status of the included participants (30). The included participants were: students (31), employees in a factory (28), staff of a training and research hospital (27), and nurses (30). Several non-inclusion/exclusion criteria were applied. They were related to habits [e.g., smoking (26, 28), alcohol-use (26), addiction to caffeinated beverages (26)], medication-use (26, 27, 29), some health complaints [e.g., sleep complaints (26)], acute diseases [e.g., upper respiratory tract infections (27), severe infections (31)], chronic conditions [e.g., unhealthy teeth or mouth (28), oral diseases (28, 31), internal diseases (28), endocrine diseases (29), diabetes mellitus (29, 30), metabolic disorders (30), cardiovascular diseases (29, 30), hearing impairment (30), headache (30), psychiatric shock (30), unspecified (27)], previous surgeries [e.g., bariatric surgery (29), head surgery (30)], pregnancy (29), weight management program (29), and job experience <1 year (30). Only one study highlighted that no participant practiced fasting as routine and voluntary rituals before the month of Ramadan (29). In one study (29), participants were asked to continue their regular diet during non-fasting hours, and not to alter their habitual physical exercise levels BR or DR. Participants’ ages varied from 24.2 ± 2.3 (31) to 59 (27) years. Participants’ weight and body mass index were reported in two (27, 29) and three (26, 27, 29) studies, respectively.

Eight different saliva parameters were evaluated: SFR (31), glucose (31), melatonin (26), cortisol (29, 30), immunoglobulin A (IgA) (27), uric-acid (28), alkaline phosphatase (ALP) (28), and aspartate amino-transferase (AST) (28). The numbers of saliva sampling were one (27, 28), two (29, 30), and three (26, 31). Different times of saliva sampling were chosen. In some studies, fixed times were applied [e.g., mid-night (26), 6h00 (30), 8h00 (26), between 11h00 and 13h00 (29), 16h00 (26)]. In some other studies, a minimum of hours of fasting was needed [e.g., 6 (31), 8 (28), 12 (27)]. Four studies reported that they opted for unstimulated saliva (27–29, 31), and only two studies reported the duration of saliva collection [e.g., 2–5 (31) and 5 (27) min]. The volume of the collected saliva (in mL) was highlighted in four studies [e.g., 2 (30), 3 (28, 31), and 5 (26)]. One study omitted to report the equipment used to analyze the saliva outcomes (29). Before saliva collection, participants were asked to rinse their mouths with water (26–31) and to avoid: ***(i)*** coughing or throat clearing into the collection tube (26), ***(ii)*** consuming caffeine and substances containing melatonin or melatonin precursors (26), ***(iii)*** eating, drinking, and smoking (29, 30), ***(iv)*** brushing (30), and ***(v)*** using oral hygiene products (29).

### Impact of Ramadan Intermittent Fasting on the Salivary Flow-Rate and Saliva Metabolites

Table 3 presents the main results of the six retained studies.

**TABLE 3:** Main results of the published studies aiming to evaluate the impacts of Ramadan intermittent fasting (RIF) on saliva parameters.

First author (ref)	Data	BR			During Ramadan								AR
					Period 1			Period 2	Period 3			Period 4	
Bahammam (26)					1st week of R				3rd week of R				**–**
	Timing	Midnight	8 a.m.	16 a.m.	Midnight	8 a.m.	16 a.m.	–	Midnight	8 a.m.	16 a.m.	–	–

	Mel*^a^*	18.1 ± 5.5	2.01 ± 1	0.62 ± 0.37	5.9 ± 8.0*	1.2 ± 1.1	0.14 ± 0.1*	–	4.1 ± 7.0^†^	3.9 ± 2.7	0.21 ± 0.1^†^	–	–
	Main aim	To assess the effect of RIF on sleep architecture, daytime sleepiness and the circadian cycle of Mel level											
	Conclusion	Midnight: Mel level has a flatter slope and a significantly lower peak for periods 1 and 3 compared to BR (BR > period 1 and BR > period 3).											
		16 a.m.: significant decrease of Mel from baseline for BR vs. period 3 and BR vs. period 1. 8 a.m.: no significant difference between BR vs. period 1											
		and BR vs. period 3. Although Mel keeps the same circadian pattern during Ramadan, its level drops significantly from baseline.											
Sariri (31)		–			R: 1–9 days			R: 10–20 days	R: 21–29 days			–	7th day after-R
	Glu*^a^*	–			54.5 ± 0.74^α^			58.8 ± 1.25^β^	63.6 ± 9.43^*W*^			–	68.5 ± 1.22
					(decrease by 25 ± 2% compared to controls)				(increase by 17 ± 2% compared to controls)			–	
	SFR	0.08–1.4			NR (10% decrease in Ramadan)							–	
	Main aim	To evaluate the influence of RIF on the level of Glu in the saliva of healthy individuals											
	Conclusion	An important decrease in salivary Glu occurred during period 1 followed by rises in periods 2 and 3. Salivary Glu											
		decreased/decreases during fasting, mainly at the beginning of the month compared with non-fasting period.											
Develioglu (27)		1 week before			–			–	–			25th day of R (last week of R)	–
	IgA*^a^*	11.15 ± 6.82			–			–	–			8.98 ± 6.85^§^	–
	Main aim	To investigate the effects of RIF on serum concentrations of IgG and IgM, and salivary IgA concentrations											
	Conclusion	Salivary IgA decreased/decreases significantly during Ramadan compared to BR.											
Khaleghifar (28)		1 day BR			–			–	15th day of R			–	–
	UA*^c^*	4.86			–			–	3.18^†^			–	–
	ALP*^c^*	14.51			–			–	17.47^†^			–	–
	AST^ c^	26.33			–			–	19.66^†^			–	–
	Main aim	To identify the influence of RIF on saliva of healthy individuals											
	Conclusion	ALP significantly increased/increases in period 3. UA and AST significantly decreased/decreases in period 3 compared with BR.											
Al-Rawi (29)		1 week BR			–			–	–			28 day of R	–
	Cor*^a^*	2.2 ± 0.40		–	–	–		2.1 ± 0.40^§^	–
	Main aim	To examine the effect of RIF on daytime levels of ghrelin, leptin, Mel, and Cor hormones in a group of overweight and obese participants							
	Conclusion	No salivary Cor levels changes during fasting compared to BR.							
Dehagi (30)		1 week BR				3rd week of R			
	**Timing**	**Morning**	**Evening**			**Morning**	**Evening**		
	Cor M*^b^* H_1_	1.41 (0.12–2.02)	0.86 (0.11–1.00)	–	–	1.61 (0.52–2.62)	1.28 (0.43–1.09)	–	–
	Cor M*^b^* H_2_	1.16 (0.81–2.43)	0.75 (0.11–0.91)			1.55 (0.83–2.46)	1.11 (0.71–1.77)		
	Cor F*^b^* H_1_	1.54 (0.32–2.31)	0.94 (0.10–1.02)	–	–	1.78 (0.62–2.91)	1.04 (0.35–1.42)	–	–
	Cor F*^b^* H_2_	1.54 (0.32–2.31)	0.83 (0.10–0.98)			1.64 (0.44–2.53)	1.53 (0.88–2.18)		
	Cor*^b^*	–	0.81 (–0.1 to 1.13)			–	1.32 (0.29–2.32)^†^		
	Main aim	To investigate the combined effects of noise exposure and RIF on salivary Cor levels in nurses							
	Conclusion	Salivary Cor increased/increases during fasting when it was/is combined with noise as another stress factor. Contradictory results: BR vs. period 3											

*ALP, alkaline phosphatase (U/L); AR, after-Ramadan; AST, aminotransferase (U/L); BR, before-Ramadan; Cor, cortisol (pg/mL); F, female; Glu, glucose (mg/100 ml); H, hospital; Ig A, immunoglobulin A (mg/dl); M, male; Mel, melatonin (pg/ml); R, Ramadan; SFR, salivary flow-rate (ml/min); UA, uric-acid (mg/100 ml). Data were:*

*^a^Mean ± SD;*

*^b^Mean (95% confidence interval);*

*^c^Mean. P-value < 0.05.*

*BR vs. period 1 (Bahammam).

*^†^BR vs. period 3 (Bahammam, Khaleghifar, Dehagi).*

*^§^BR vs. period 4 (Develioglu,Al-Rawi).*

*^α^AR vs. period 1 (Sariri).*

*^β^AR vs. period 2 (Sariri).*

*^W^AR vs. period 3 (Sariri).*

#### Salivary Flow-Rate

The only study evaluating the SFR reported its decrease by 10% DR compared to controls (31).

#### Salivary Hormones: Melatonin and Cortisol

Khaleghifar et al. (28) reported that melatonin keeps the same circadian pattern DR, but its level drops significantly from baseline. At midnight, melatonin level has a flatter slope and a significantly lower peak in the first and the third weeks of Ramadan compared to BR. At 8 a.m., there is no significant difference between BR and the first or third weeks of Ramadan. At 16 a.m., there is a significant decrease of melatonin from baseline for BR vs. the first or third weeks of Ramadan.

Regarding salivary cortisol levels, studies reported different results (29, 30). One study reported no change in salivary cortisol levels DR compared to BR (29). Another study reported that RIF has a significant effect on salivary cortisol secretory levels (30). The latter increases during fasting when it is combined with noise as another stress factor (30).

#### Salivary Metabolic and Immunologic Data

Sariri et al. (31) reported a significant decrease in salivary glucose during the first 10 days of Ramadan (by 25% compared to controls), the 10–20 days of Ramadan, and 21–29 days of Ramadan (by 17% compared to controls). Khaleghifar et al. (28) reported that compared to BR, on the 15th day of Ramadan, ALP significantly increases, and uric-acid and AST significantly decrease. Develioglu et al. (27) noted that salivary IgA decreases significantly during the last week of Ramadan compared to BR.

## Discussion

The present systematic review included six studies, all having a high score of bias (26–31). In these studies, eight saliva parameters were evaluated (SFR, melatonin, cortisol, glucose, IgA, uric-acid, ALP, and AST). The main results were: ***(i)*** the SFR decreased by 10% DR in fasting participants compared to controls (31), ***(ii)*** the circadian pattern of melatonin was unchanged DR, but melatonin level dropped significantly from baseline (28), ***(iii)*** the salivary cortisol levels were unchanged DR compared to BR (29), or increased DR (30), ***(iv)*** the salivary glucose levels were decreased DR (31), ***(v)*** compared to BR, on the 15th day of Ramadan, ALP significantly increased, and uric-acid and AST significantly decreased (28); ***(vi)*** the salivary IgA decreased during the last week of Ramadan compared to BR (27). All the retained studies were heterogeneous and had several methodological differences. This heterogeneity limited the ability of the present review to perform any data synthesis *via* meta-analysis. It also challenged the researchers’ ability to identify trends in the data. Research reports in this area are few and they were almost limited to the changes of glucose concentrations in plasma (31). To the best of the authors’ knowledge, this is the first systematic review investigating the effects of RIF on SFR and saliva parameters.

### Impacts of Ramadan Intermittent Fasting on Salivary Flow-Rate

SFR decreased by 10% DR (31). DR, the lack of gustatory stimulation decreases the stimulation of salivary glands, therefore, SFR may decline. The autonomic nervous system controls SFR and the secretion of various salivary compounds (32). Stimulation of this system induces modifications in salivary secretions and SFR (33). In Ramadan, sedentary activity with minimal orofacial movement and metabolism slowing down in body tissues cells, including oral cavity cells, may explain the low stimulation of the autonomic nervous system (28). This hyposalivation can cause malodor, especially DR (34). Since saliva works to moisten the mouth, to neutralize acids produced by plaque, and to clean bacteria and food particles from the mouth, any salivary modifications create a suitable environment for aerobic and anaerobic bacteria that coat several sites in the oral cavity, notably the dorsum of the tongue (35). Overall, it has been shown that oral microflora modifications taking place DR may lead to malodor, even if other factors are involved (36).

### Impact of Ramadan Intermittent Fasting on Salivary Hormones: Melatonin and Cortisol

Melatonin in saliva or plasma is an indicator of the timing of the circadian clock (37). According to Bahammam (26), the sleep hormone follows the same circadian rhythm both BR and DR. This means that melatonin secretion is low during the daytime, while the highest levels are released at night, but its level drops significantly from baseline (26). This variation may be due to the sleep habits modification DR (36). Nevertheless, this outcome should be considered with caution because of the small sample size in the study (*n* = 8) (26).

Cortisol is a hormone produced by the adrenal glands (38). Cortisol plays an essential role in balancing blood glucose and releasing sugar from the body’s stores in response to increased energy demands (39). Cortisol has an important role in the metabolism of fats and proteins as well as in the circadian rhythm regulation (38). This hormone is usually measured in the morning (7–9 a.m.) because it reaches a peak at this time (40). DR, external sources of glucose are reduced due to fasting. Consequently, salivary glucose concentration drops significantly (31). Thus, we can “speculate” that cortisol levels in saliva may rise to regulate glucose levels, however; the latter mechanism is not that straightforward and has to be elucidated by further research.

Dehagi et al. (30) reported that when participants are exposed to RIF and noise, which is another stress source, salivary cortisol levels increase. In addition to its glycemic effects, cortisol is also liberated during the stress periods in order to allow the body to adapt to an emotional or physical shock by mobilizing additional energy sources. The contradictory results of the studies of Al-Rawi et al. (29) and Dehagi et al. (30) may be due to methodological reasons, notably the study design and population, and the lack of information about the timing and duration of sleep in one study (30). It should be highlighted that many people in various Islamic countries may change their sleep rhythm during the Holy month. Indeed, their nighttime sleep duration is reduced compared to non-fasting days (41), in addition to the dietary patterns’ changes (42).

### Impact of Ramadan Intermittent Fasting on Salivary Metabolic and Immunologic Data

Alkaline phosphatase and aminotransferase are usually measured together to investigate the hepatic, cardiovascular, and renal functions (43). ALP is a protein produced by various cell types (e.g., polymorphonuclear leukocytes, osteoblasts, macrophages, and fibroblasts) within the alveolar bone and/or the salivary glands (44, 45). ALP can be a salivary biomarker of periodontal diseases and caries (46), as it interferes in the balance of the remineralization-demineralization cycle since it is primarily involved in calcium and phosphate binding (47). It seems that the function of ALP relatively depends on the salivary pH and buffering capacity (48). Khalighefar et al. (28) reported that ALP rebounds during the middle of Ramadan compared to BR. Although ALP increase may suggest much more susceptibility to dental caries and/or oral diseases, it is believed that this fluctuation is not so critical to lead to an illness. AST is an enzyme involved in the metabolism of several tissues and organs (49). Khaleghifar et al. (28) indicated that AST activity in fasting volunteers decreases significantly DR. This decrease can be related to the fact that fasting reduces the metabolism of body tissues cells, including oral cavity cells, thus leading to reduced SFR during fasting (28). Uric-acid is the ultimate product of the metabolic breakdown of purines, which are the nitrogenous bases in DNA and RNA (50). It is involved in healing and defense (50). Khaleghifar et al. (28) reported that uric-acid decreases DR since the metabolism is reduced (28). In contrast, several studies have shown that blood uric-acid increases during RIF (51–54). According to studies reported in the literature, despite the shifts in metabolic interactions among the organs producing uric-acid, AST or ALP, we cannot conclude on the effects of RIF on these enzymes because of the scarcity of these studies in addition to the limitations of the unique retained study investigating those parameters (28).

Salivary glucose DR plunges from baseline, especially in the first 10 days (31). First, this is expected because of food restriction for 4 weeks. Secondly, this fact is interesting and beneficial for oral health. Actually, both cariogenic bacteria and *Candida* use glucose for their development and survival (55, 56). This dysbiosis enhances the proliferation of these bacteria and dental biofilm development (46, 57). A recent study investigated the effect of different salivary glucose concentrations on dual-species biofilms of *Candida albicans* and *Streptococcus mutans* (58). The authors reported that higher salivary glucose increases counts of *Candida albicans* (58). It is possible that the higher levels of IgA detected in saliva BR can be attributed to the greater colonization of the oral cavity by *Candida albicans* due to the higher salivary glucose levels during that period compared to DR.

Salivary IgA has an important role in mucosal immunity. Its levels increase in case of oral mucosa infection, such as candidiasis. It allows inhibiting the adherence of *candida* to epithelial cells (59, 60). In contrast, the decrease in those salivary IgA levels does not necessarily suggest that the participant is more susceptible to oral infection onset, since a salivary IgA concentration threshold is absent (27). Subsequently, authors suggested that RIF results in neither severe immunological disturbances nor adverse impact on health (27). Some remarks related to the usefulness of salivary IgA in real practice should be highlighted. First, there are some concerns regarding the usefulness of salivary IgA as a biomarker in the detection of respiratory tract infection due to lack of reproducibility, low specificity, and sensitivity (61). Secondly, there are conflicting data in the literature regarding salivary IgA levels induced by exercise, with some studies reporting a decrease whilst others have reported an increase or no change (62). Thirdly, previous studies have reported a decrease in systemic IgA levels without leading to an increase in infection (63). Fourthly, exposure to pathogenic microbes may be reduced DR, possibly due to consumption of more fresh foods DR compared to other months (64). It is possible that oral health and microbial exposure from foods are poorer BR, which may explain the higher IgA levels detected in the saliva DR (65). In this context, a recent study involving mice reported that oral colonization by *Candida albicans* increases IgA production (65). Another study suggested that an increase in salivary IgA is an attempt by the immune system to counter the accumulation of microorganisms (64). Considering the aforementioned studies (63–65), the decrease in IgA levels DR may reflect a lower microbial colonization of the oral cavity DR. This is plausible since the number of hours when the mouth is exposed to foods and beverages is reduced DR compared to other periods when one considers the number of hours spent fasting and sleeping.

Overall, it seems that fluctuations in salivary parameters in Ramadan are not as significant as blood changes. These alterations are not enough to cause diseases in healthy participants. Nevertheless, we believe that further studies using other salivary biomarkers are needed in order to investigate correlations with the risk of oral disturbances or infections, such as caries, malodor, periodontal disease, or candidiasis in Ramadan.

In view of the absence of evidence about the impacts of RIF on oral health, we recommend the following four advices for people observing Ramadan: ***(i)*** adopt a well-balanced diet with sufficient hydration before *Sahur* and after *Iftar*; ***(ii)*** brush teeth, at least after *Iftar* and just after *Sahur*, before the dawn; ***(iii)*** rinse mouth without swallowing water for a better biofilm control and reduction of halitosis; and ***(iv)*** take care of the oral cavity, particularly for patients with chronic systemic diseases, especially with metabolic disorders (e.g., diabetes mellitus) in order to avoid the progression of a preexistent pathology (e.g., periodontal disease, dental caries). Finally, it is recommended that dentists carry out “dental procedures” with special precautions [e.g., administer intramuscular or trans-dermal treatment instead of oral agents] (36).

### Discussion of Methodology

According to the JBI critical appraisal tool, precisely the checklist for cohort studies, the methodological quality is considered as “low.” In fact, no study succeeded to get the average score and items related to “confounding factors” and “sample size calculation.” Moreover, “salivary collection methods” were not reported in any of the six retained studies (Table 1). First, non-inclusion of a non-fasting control group can be considered as a “bias” since the variations in the assessed parameters cannot be exclusively attributed to RIF. However, it is important to note that including non-fasting participants is still problematic, due to religious considerations in Muslim countries. For that reason, the non-fasting control groups could be the participants themselves outside the Ramadan period (e.g., BR and/or AR). Given the circumstances of the Ramadan observance, and for practical reasons, the authors think that is more feasible and easier to control the parameters than to arrange a separate group of participants who do not observe Ramadan. Secondly, selecting participants by a convenience sample may be considered as a major confounding factor (66). Convenience sampling is a type of non-probability sampling methods based on the judgment of the investigator (66). Its low cost and comfort of use make it an easy choice for investigators. Nevertheless, it can lead to under/over representation of specific groups inside the sample (66). Thus, it may be impossible to make generalizations in the whole population. For these reasons, convenience sampling should be treated with caution. Thirdly, calculation of an optimal size is a crucial point since it helps avoid an inadequate power to detect statistical effects (67). Using few participants in a study may lead to lower “precision” in findings. A large sample size is, however, expensive and exposes more participants to procedures (67). Fourthly, the procedure of saliva collection was not well-described (Table 1). In fact, it is very important to standardize saliva sampling in order to make comparison between studies possible. Since saliva collection should be made at least one time DR, unstimulated saliva might be preferred. In fact, stimulated saliva must be collected by chewing sterile paraffin (68). Then, a minimum duration for sufficient saliva collection may be defined to ensure efficient analysis.

Additional limitations should be highlighted. For example, information about the season, the average ambient pressure, and/or the average ambient humidity was lacking in the included studies (Table 1). The average ambient temperature as well as the fasting duration were mentioned in some studies (26–29) (Table 1). Consequently, both climatic conditions and geographical locations strongly influence RIF (69). Also, the inclusion of patients with obesity (i.e., body mass index ≥ 30 kg/m^2^) may be considered as a limitation. In fact, a lower SFR was observed among obese compared to non-obese participants (70, 71). In addition, the inclusion of females and old participants could complicate the interpretation of saliva parameters (72, 73). Indeed, Mahesh et al. (72) reported significant changes in the pH and the buffer-capacity in post-menopausal females’ saliva compared to regularly menstruating ones. Besides, it is known that females do not fast all the month of Ramadan. Subsequently, the comparison with males may not be valid because they are not exposed to the same fasting period. With regard to age, changes in salivary pH, buffering-capacity, calcium, and proteins concentrations were reported (73). Finally, the number of evaluation sessions was heterogeneous. Therefore, saliva collection should be performed at least three times as follows: BR (e.g., 1 week BR), DR (e.g., during the last 7–10 days of Ramadan), and AR (e.g., 7–10 days AR). In future studies aiming to evaluate the effects of RIF on oral health, three important points should be reported. The first is related to the practice of fasting as a routine (e.g., some Muslims fast on Mondays and Thursdays during all the year). The inclusion of some participants who practice this ritual may influence some saliva parameters. The second point concerns the chewing stick, called “*Miswak*,” which is widely used in some Arab states of the Persian Gulf (74, 75). In fact, it seems that “*Miswak*” use increases SFR (76). The third point concerns the hydration status. The role of the hydration status BR and DR were not considered in the six retained studies and the differences observed in the concentrations of the different metabolites may be partly due to the hydration status, which can alter the salivary composition and SFR (77). The six studies involved in this review did not adjust the concentrations of the different salivary biomarkers before comparing the data obtained BR and DR. Therefore, in the future, it would be interesting to see if the differences observed are still present after adjusting the metabolites by factors, such as total protein content, saliva osmolality, SFR, and saliva secretion rate (78).

The critical limitation of this Systematic Review is our inability to make a strong clinical case for the impacts of RIF on saliva parameters. Table 4 summarizes some recommendations for designing future studies aiming to investigate the impacts of RIF on saliva parameters. It is recommended that researchers assess the antimicrobial, anticancer, and wound healing properties of fasting saliva (collected just before *iftar*) and compare it with non-fasting saliva. Moreover, it would be great to compare the fasting saliva proteome with the non-fasting saliva, and to see if the fasting saliva can be a source of novel peptides that display health benefits (79). This will address the “myth/superstition” in medieval Europe where fasting saliva was used as a medicine (80, 81).

**TABLE 4:** Some recommendations for designing future studies related to the impact of Ramadan intermittent fasting on salivary parameters.

Issue	Authors are encouraged to:
General remarks	⋅Report information about the following points: season of Ramadan, ambient temperature and humidity during the study period, elapsed fasting time, and number of fasting days during the Ramadan month. ⋅Report the exact timing of the saliva samples.
Study protocol/design	⋅Opt for a cohort design. ⋅Include a non-fasting control group, if possible. ⋅Select participants using a probability sampling method. ⋅Perform at least three evaluation sessions: before-Ramadan (e.g., 1 week), during-Ramadan (e.g., during the last 7–10 days of Ramadan) and after-Ramadan (e.g., 7–10 days AR).
Population characteristics	⋅Avoid the combination of males and females in one sample. ⋅Systematically report the following confounding factors which interact with saliva parameters: age, smoking status, alcohol drinking, hydration status, total fluid intake (coffee, tea, juice, etc.), dietary habits, sleeping habits, teeth brushing, physical activity, obesity, *Miswak* use, fasting ritual. ⋅Spitting out or not (some people do not want to swallow their saliva, mistakenly thinking that it will break their fast). ⋅Determine how often the mouth is rinsed with water (some people avoid rinsing their mouth with water thinking this will break their fast).
Saliva collection and analysis	⋅Use standardized and reliable methods of saliva sampling. ⋅Use standardized methods of biological analysis (e.g., concentration of biomarkers should be adequately adjusted by factors, such as osmolality, total protein concentration, saliva flow-rate, and saliva secretion rate). ⋅Opt for unstimulated saliva rather than stimulated saliva. ⋅Report the normal range of saliva parameters. ⋅Adjust the metabolites by factors, such as total protein content, saliva osmolality, saliva flow-rate, and saliva secretion rate.
Sample size and statistical analysis/methods	⋅Calculate the sample size. ⋅Report and interpret the effect size measurement (if needed). Clearly distinguish the “clinical” significance approach from the “statistical” significance approach.

## Conclusion

There is a general trend toward a decrease in SFR and a decrease in the content of the majority of the biomarkers investigated, with the exception of ALP and uric-acid. These changes cannot be easily attributed to any single factor, especially because of the lack of information on the hydration status, dietary habits, physical activity, and hygiene habits. Although the findings of this systematic review are interesting, scientific evidence should be interpreted carefully because studies of the impact of RIF on saliva parameters are scarce. This is mostly due to the lack of accurate methodological details or variations in the investigated saliva parameters and the employed methodologies. Furthermore, the authors have provided some recommendations for designing future studies related to the impact of RIF on salivary parameters.

## Data Availability Statement

The original contributions presented in the study are included in the article/supplementary material, further inquiries can be directed to the corresponding author.

## Author Contributions

AB, MK, and HB performed bibliographic research, collected published manuscripts, and helped to draft the manuscript. NB helped draft the manuscript. All authors read and approved the final manuscript.

## Conflict of Interest

The authors declare that the research was conducted in the absence of any commercial or financial relationships that could be construed as a potential conflict of interest.

## Publisher’s Note

All claims expressed in this article are solely those of the authors and do not necessarily represent those of their affiliated organizations, or those of the publisher, the editors and the reviewers. Any product that may be evaluated in this article, or claim that may be made by its manufacturer, is not guaranteed or endorsed by the publisher.

## Abbreviations

ALP: alkaline phosphatase
AR: after-Ramadan
AST: aspartate amino transferase
BR: before-Ramadan
DR: during-Ramadan
IgA: immunoglobulin A
JBI: Joanna Briggs Institute
RIF: Ramadan intermittent fasting
SFR: salivary flow-rate.
